# The microenvironmental mechanism of postoperative recurrence in cervical spondylotic myelopathy: regulation by the glial scar–inflammation axis

**DOI:** 10.3389/fneur.2026.1774739

**Published:** 2026-03-30

**Authors:** Yibing Sun, Qingguo Zhang

**Affiliations:** 1 Clinical Medical College of Shandong Second Medical University, Weifang, Shandong, China; 2 Jinan Central Hospital Affiliated to Shandong First Medical University, Jinan, Shandong, China

**Keywords:** cervical spondylotic myelopathy, glial scar, immune microenvironment, inflammation axis, postoperative recurrence

## Abstract

Cervical spondylotic myelopathy (CSM) is a severe degenerative spinal disorder caused by cervical spinal stenosis due to cervical degeneration, which compresses the spinal cord. Patients in the mid-to late-stages of the disease frequently undergo surgical treatment; however, some may still suffer from persistent sensorimotor dysfunction, inadequate pain relief, and surgery-related complications. Although substantial progress has been achieved in comprehending the pathology of CSM in recent years, the postoperative pathological mechanisms remain poorly understood, particularly the specific molecular mechanisms influencing the development of complications. Traditional research has focused on mechanical compression caused by herniated material, neglecting the potential adverse effects of postoperative immune microenvironment imbalance in the spinal cord. Current studies suggest that the glial scar–inflammation axis, which is triggered by abnormal activation of neural immune cells (glia) and peripheral immune cells (e.g., Th17 cells and neutrophils) and their interactions—may serve as a key factor contributing to poor postoperative outcomes and disease recurrence. This review summarizes the recent advances in the biology and pathology of the glial scar–inflammation axis following conventional surgical treatment for CSM, as well as innovative therapeutic strategies, such as stem cell transplantation. It aims to provide new insights and directions for future research on postoperative complications and their treatment in CSM.

## Introduction

Cervical spondylotic myelopathy (CSM) is the most severe form of degenerative cervical myelopathy (DCM) and stands as the most prevalent chronic neurological disorder among individuals aged 55 and above (1–3). The primary pathological characteristic of CSM involves cervical degenerative changes, such as disc herniation, osteophyte formation, ligamentum flavum hypertrophy, and ossification of the posterior longitudinal ligament. These changes lead to spinal canal stenosis, resulting in prolonged or sustained spinal cord compression and, ultimately, spinal cord injury (SCI). Clinical manifestations encompass limited limb mobility, sensory deficits, positive pathological reflexes, gait abnormalities, and bladder-bowel dysfunction. Without timely intervention, irreversible spinal cord damage, such as paralysis or even death, might ensue (4). Treatment options for CSM include conservative approaches (e.g., cervical immobilization, medication, lifestyle modifications, and physical therapy) and surgical decompression, with the latter being the primary method for alleviating CSM-related symptoms. Although most patients experience improvement postoperatively, 11–38% develop complications, and 5–15% exhibit stalled neurological recovery, recurrence, or disease progression (5). Several prognostic factors influence surgical outcomes, with the most significant being symptom duration, preoperative neurological status, effective canal diameter, number of compressed levels, intrinsic spinal changes (assessed via preoperative Magnetic Resonance Imaging [MRI]), and other clinical-radiological features (6, 7). Sadasivan et al. demonstrate that symptom duration exceeding 18 months before diagnosis correlate with poor outcomes (8). In a clinical trial, Zoher Ghogawala et al. report that ventral surgery carries a significantly higher risk of complications—including dysphagia, new neurological deficits, reoperation, and readmission within 30 days—compared to dorsal approaches (9). Chagas et al. also evaluate the benefits of anterior decompression and fusion (ADF) in CSM patients, revealing that 64.1% improve on the Nurick scale, 33.3% remain unchanged, and 2.6% worsen. Additionally, CSF leakage, hoarseness, dyspnea, and dysphagia are more commonly associated with the anterior approach (10). Moreover, patients under 60 years of age are more likely to benefit from surgery than those over 60 (11). Furthermore, improper use of cervical braces, insufficient functional exercise, chronic poor posture (e.g., forward head posture), trauma, or heavy physical labor can lead to repeated spinal compression, thereby accelerating disease recurrence.

Clinical research on the CSM recurrence has primarily focused on surgical technique improvements (e.g., interbody fusion methods) and optimization of decompression extent. This research has been confined to a dual “mechanical compression–decompression” model, which merely enlarges the spinal canal and relieves physical pressure (12). Current prognostication relies on factors such as age, symptom duration, preoperative neurological status, and imaging findings (13, 14). Postoperative canal volume recovery is assessed by imaging, for instance, using MRI signal intensity (T2 hyperintensity) to evaluate neurological recovery and changes in spinal cord nucleus signals (15). However, these approaches and evaluations fail to consider the spinal cord heterogeneity or postoperative microenvironmental changes. This explains why patients with similar compression degrees may exhibit vastly different neurological outcomes. Studies have indicated that even after decompression, glial scar formation and chronic inflammation within the spinal cord can inhibit axonal regeneration and myelination. Thus, non-mechanical stimuli, such as glial scarring, inflammation, and oxidative stress, may contribute to disease progression post-treatment. Imaging (e.g., MRI) and autopsy studies consistently show dense glial and fibrotic scars in chronic patients. These structures form and persist long after acute mechanical injuries such as compression, contusion, transection (16). Pure mechanical decompression cannot fully explain the variability in postoperative neural repair. Disordered local immune microenvironments post-SCI may be one of the reasons for recurrence (17).

Local immune microenvironment imbalance refers to a persistent pathological state wherein abnormal activation of immune cells (e.g., microglia, macrophages), dysregulation of pro-inflammatory and anti-inflammatory factors, and disrupted cellular crosstalk continue to exist even after surgical relief of mechanical compression. Similar strategies are being employed in multiple sclerosis (MS) treatment and rheumatoid arthritis, though clinical trials in CSM are still in their early stages (18, 19). This process originates from the dysregulated interactions between central glial cells and peripheral immune cells. Aberrant glial activation induces the formation of central scars and amplifies inflammatory cascades, while immune-glial interactions suppress reparative immune responses, thereby hindering neural repair (20–24). Moreover, clinical cohort analyses have revealed that patients with persistently elevated local levels of IL-1β, IL-6, and TNF-*α* postoperatively exhibit significantly lower rates of neurological recovery compared to those with normal cytokine levels. Thus, high IL-6 and TNF-α levels are significantly associated with poor postoperative neurological recovery in CSM patients. Additionally, clinical data indicate that preoperative serum TNF-α levels positively correlate with postoperative cognitive decline, with a sensitivity of 82.7% for predicting poor recovery (4, 23, 24).

Additionally, animal models serve as an indispensable bridge between *in vitro* testing and human clinical trials, providing a sophisticated living system to evaluate pathological mechanisms, tissue responses, and therapeutic efficacy under controlled conditions (25, 26). Studies demonstrate that targeted interventions against glial scar formation, such as using chondroitinase ABC to degrade chondroitin sulfate proteoglycans, or downregulating inflammatory factors with anti-IL-17A antibodies, can effectively promote functional recovery in experimental SCI models (18, 27, 28). These findings suggest that dynamic imbalance in the postoperative immune microenvironment may be a key factor in CSM recurrence, indicating that the glial scar–inflammation axis could be a potential interventional target. Therefore, this review elucidates the interaction mechanisms between glial scarring and inflammation from the perspective of the immune microenvironment, aiming to provide new research directions for improving postoperative outcomes in CSM patients.

## Formation of glial scar

### Etiology of glial scar formation

Tran et al. emphasize that primary mechanical compression and surgical trauma induce glial scar formation post-CSM surgery. The glial scar is a central factor in regeneration failure post-SCI, and therapeutic strategies targeting its inhibitory components are clinically crucial for improving long-term outcomes (29). The neuroglial scar is a dense boundary formed by reactive astrocytes, microglia, and NG2 glia after central nervous system (CNS) injury, which isolates severely damaged areas (30). Mechanical compression causes local ischemia and hypoxia, leading to oxidative stress and mitochondrial dysfunction. The reduced mitochondrial membrane potential triggers cytochrome c release, ultimately activating caspase cascades and inducing neuronal apoptosis (31). Prolonged compression also damages spinal microvascular endothelial cells, further compromising the blood-spinal cord barrier (BSCB) (32). Surgery relieves physical compression via durotomy and laminectomy, but it also causes SCI, prompting the release of plenty of inflammatory mediators (e.g., substance P, bradykinin) and damage-associated molecular patterns (DAMPs; e.g., HMGB1, glutamate, ATP). These substances activate nearby glial cells and exacerbate local microenvironment disruption (33, 34). Injured microglia can differentiate into the M1 pro-inflammatory phenotype within hours, secreting cytokines such as IL-1β and TNF-*α* (21). Within 72 h, astrocytes proliferate, reorganize the extracellular matrix (ECM), and form a dense scar primarily composed of chondroitin sulfate proteoglycans (CSPGs), laminin, and collagen types I and III. Astrocytes enlarge and migrate around the severely injured area, interweaving to form the main component of the glial scar at the injury core (35–37).

### Molecular characteristics of glial scar

**Figure 1:**
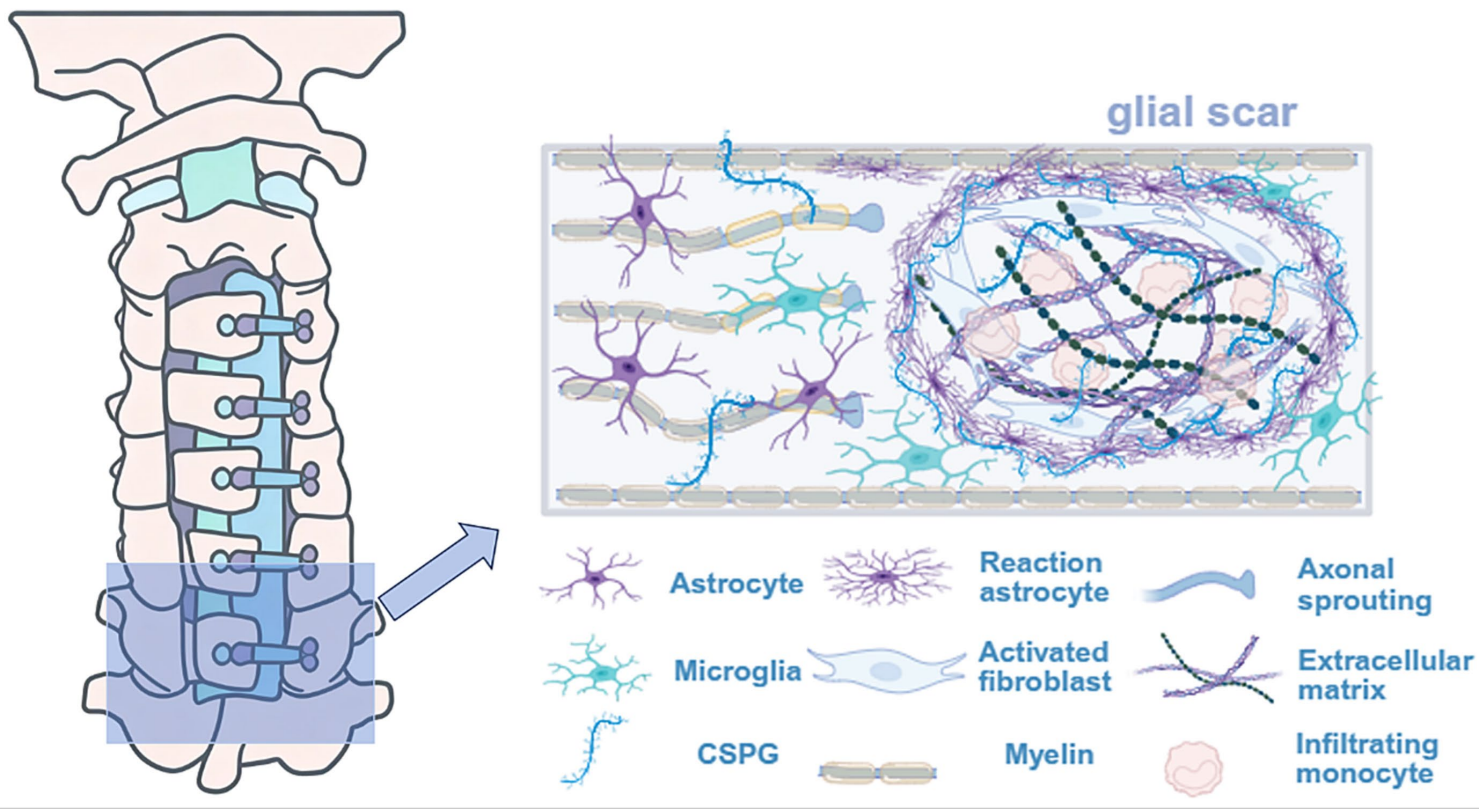
In the repair of chronic cervical spondylotic myelopathy injury, the lesion core contains a fluid-filled cystic cavity enriched with activated microglia and macrophages. This core is bordered by reactive astrocytes forming a glial scar, along with fibroblasts. Reactive astrocytes secrete CSPGs into the extracellular matrix, which contributes to glial scar formation. The glial scar sequesters the injury core and limits the spread of inflammation, while simultaneously inhibiting axonal regeneration and attracting inflammatory cells.

The neuroglial scar is characterized by a dynamic imbalance between cellular and non-cellular components (38). At the cellular level, activated astrocytes upregulate glial fibrillary acidic protein (GFAP) and vimentin, forming a dense envelope around the lesion core (39). The lesion area is abundant in fibroblast-like cells that secret TGF-*β*, promoting collagen deposition (40–42). On a molecular level, CSPGs within the scar serve as the primary molecular barrier to neural regeneration. CSPGs bind to LAR family receptors on axons, activating the RhoA/ROCK pathway and inhibiting axonal growth cone extension (43). An imbalance between tissue inhibitor of metalloproteinases (TIMPs) and matrix metalloproteinases (MMPs) in the scar matrix results in excessive ECM deposition, which not only hinder the neural tissue repair but also exacerbate secondary injury and chronic neurological deterioration (44). The role of the neuroglial scar is dualistic: within 1–2 weeks post-surgery, it isolates the injury core, limits the inflammation spread, and prevents secondary neuronal death (35). However, scar compression is closely related to progressive neurological damage postoperatively. At 3–6 months post-surgery, significant expression of ECM proteins like GFAP and CSPGs persists in the perilesional glial scar, and their expression may even increase over time, impeding regeneration (37). Beyond its physical barrier effects, scar compression alters the local ECM mechanical properties, creating a “trap” that attracts and retains inflammatory cells (45). Furthermore, inhibiting scar formation in animal models promotes axonal regeneration and motor recovery, suggesting that balancing neuroprotection and regeneration by modulating glial scar formation could help control CSM recurrence (27). The molecular mechanisms involved in the repair process of chronic cervical spondylotic SCI are illustrated in Figure 1.

### Beneficial effects of glial scar

The early glial scar plays a protective role. Post-CSM local injury is characterized by ionic imbalance, radical accumulation, glutamate excess, and the reactive oxygen species (ROS) production (46). Infiltrating peripheral immune cells and activated resident microglia can trigger excessive inflammatory responses, which may damage nearby normal tissue (47). The early glial scar confines inflammatory cells and toxic molecules to the lesion site, preventing damage to healthy spinal tissue (46). Studies show that reduced reactive astrocytes after SCI lead to impaired BSCB repair, worsened inflammation, severe demyelination, neuronal and oligodendrocyte degeneration, as well as motor deficits, indicating that early astrocyte loss may be a key cause of secondary injury (48). Genetic knockout models in adult mice with SCI reveal that preventing astrocyte scar formation, reducing scar-like astrocytes, or chronically eliminating the astrocytic scar does not spur spontaneous regeneration of sensory or serotonergic axons. For neuroglial scars, applying growth factors at the injury site yields better regenerative outcomes than preventing astrocyte scar formation (37, 49, 50). In summary, although the roles of glial scars and astrocytes post-SCI are complex, inflammation is a consistent underlying theme.

### Adverse effects of glial scar

Reactive astrocytes exert a negative impact on functional recovery during the damaged neural tissue repair in the CNS. The primary reason is that the glial scar acts as a physical barrier to axonal regeneration, preventing axons from extending into the injury site (51). Moreover, CSPGs—key chemical components of the neuroglial scar (including versican, neurocan, and brevican)—can severely restrict axonal regeneration, sprouting, and remyelination post-SCI (52–55). Takeuchi et al. find that CSPGs inhibit neurite outgrowth in the CNS *in vitro* (56). Similarly, other studies show that knocking out the key enzyme for CSPG synthesis—CSN-acetylgalactosaminyltransferase 1, or treating with the CSPG-degrading enzyme chondroitinase ABC enhances motor recovery and axonal regeneration in SCI mice (57). PTPσ is a CSPG receptor whose binding to CSPGs leads to inhibited axonal regeneration (57, 58). Intracellular peptide mimetics inhibiting PTPσ can mitigate CSPG-mediated inhibition (58). Due to these adverse effects, the mechanisms driving scar formation have been extensively studied. TGF-β increase post-SCI activates Smad signaling, promoting astrocyte proliferation and CSPG expression. These effects are inhibitable by TGF-β receptor inhibitors and paclitaxel (59, 60). Paclitaxel inhibits the Smad2/3 nuclear translocation, thereby blunting TGF-β signaling (42). Additionally, inhibiting the JAK/STAT3 and JNK/c-Jun pathways suppresses astrocyte activation and proliferation, reduces glial scar formation, and promotes functional recovery (61).

## The postoperative inflammatory cascade

**Figure 2:**
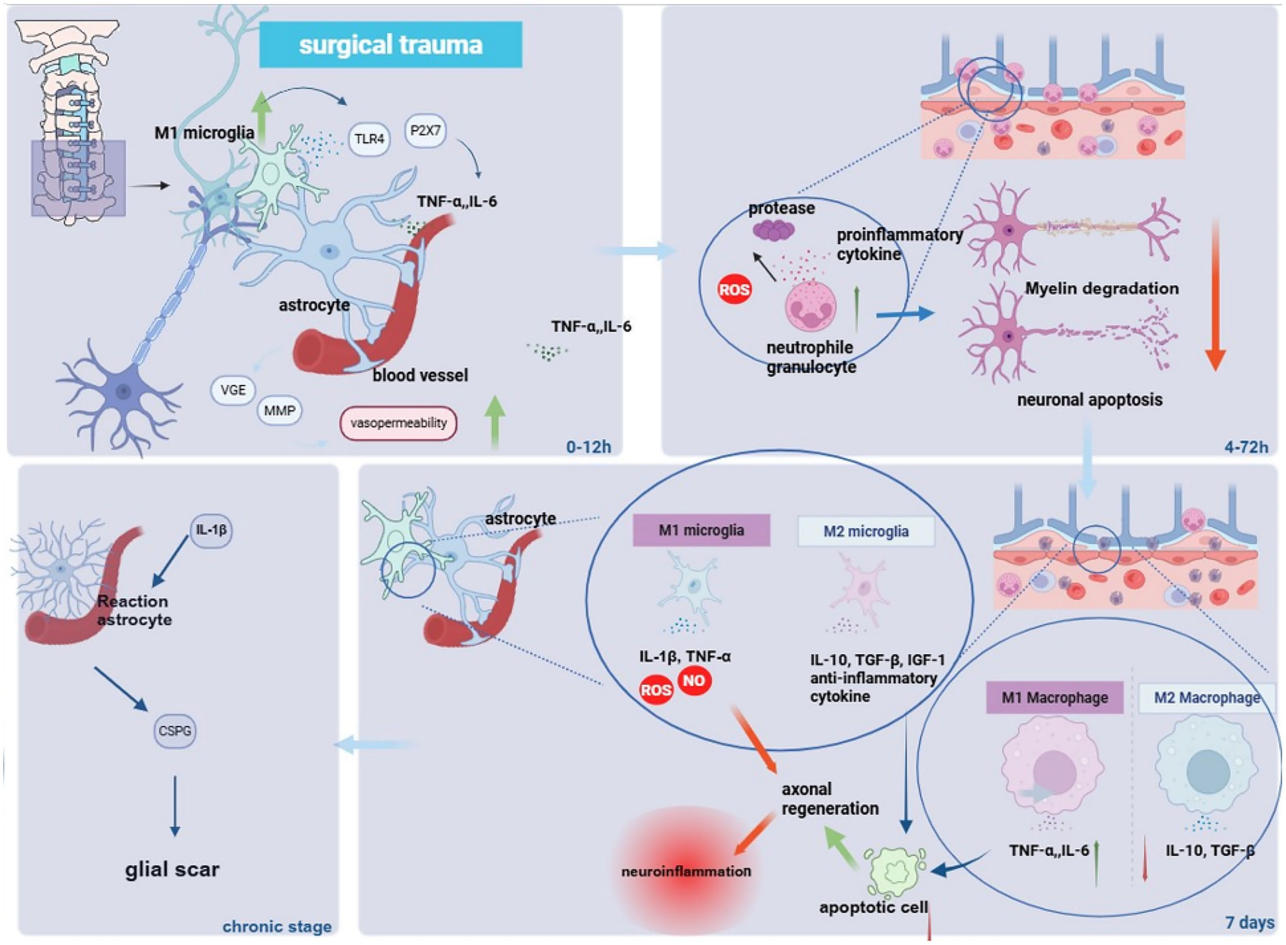
Schematic illustration of the inflammatory microenvironment driving astrocyte activation and glial scar formation in postoperative cervical spondylotic myelopathy. It summarizes the key molecular and cellular interactions within the postoperative inflammatory microenvironment that drive the pathogenic activation of astrocytes and subsequent glial scar formation, ultimately contributing to poor neurological recovery in cervical spondylotic myelopathy.

Surgical decompression, while relieving mechanical compression, inevitably inflicts trauma that initiates a complex and dynamic inflammatory cascade within the spinal cord microenvironment. This cascade is a critical determinant of postoperative outcomes in CSM. As illustrated in Figure 2, this process unfolds over distinct temporal phases, beginning with immediate cellular responses and progressing to chronic glial scar formation. Inflammation is a key component of the secondary response, directly or indirectly determining CSM prognosis. Inflammatory markers (e.g., C3, IL-6) and imaging features (T2 hyperintensity) may serve as biomarkers for CSM outcomes (62). Numerous studies find that acute inflammation helps clear tissue debris and elevate neurotrophic factor levels (63, 64), whereas chronic inflammation releases large amounts of pro-inflammatory cytokines, proteases, MMPs, and ROS, leading to inflammatory cell infiltration and exacerbating damage to surrounding healthy spinal tissue (65–67).

### BSCB disruption and microglial initiation

The BSCB, composed of endothelial cells, pericytes, and astrocytic end-feet, is intact, sealed, and structurally stable. It prevents entry of blood metabolites and neurotoxic molecules while transporting nutrients to the brain. Composed of tight junction proteins and a basement membrane, structural instability can cause BSCB hyperpermeability or rupture (29, 68). Postoperatively, the BSCB may partially mitigate the surgical pressure on the spinal cord, but mechanical stimulation from the incision can induce spinal ischemia. Damage to vascular endothelial cells within the BSCB also leads to excessive secretion of vascular endothelial growth factor (VEGF) and MMPs during circulation, degrading basement membrane collagen IV and laminin, and increasing vascular permeability. Simultaneously, DAMPs activate TLR4 and P2X7, inducing the expression of adhesion molecules on endothelial cells and promoting the adhesion and migration of peripheral immune cells (69). Microglia respond rapidly within 0–12 h, in synergy with TLR4 and P2X7 activation, secreting TNF-*α* and IL-1β to initiate inflammatory cascades and upregulate chemokines CXCL1/CXCL2 to recruit neutrophils, as depicted in Figure 2 (upper left panel).

### Neutrophil infiltration and immune cell recruitment

The compromised BSCB and the chemokine milieu (including CXCL1/2) created by activated microglia and astrocytes serve as powerful signals for the recruitment of neutrophil granulocytes from the bloodstream (Figure 2, upper right panel). This wave of neutrophils arrives at the injury site within hours (4–72 h), indicating the extent of SCI (29). Postoperatively, peripheral immune cells (e.g., neutrophils, monocytes, T lymphocytes) infiltrate the spinal parenchyma (70). Neutrophils are among the first peripheral immune cells to arrive at the injury site, reaching a peak around 24 h post-SCI (29). Studies conducted by Neirinckx and Yokota et al. have shown that invasive neutrophils can secrete leukocyte protease inhibitors to promote repair. G-CSF (granulocyte colony-stimulating factor) therapy, related to their regulation, has entered clinical trials with preliminary efficacy (71, 72). However, neutrophils also release a large amount of pro-inflammatory cytokines, proteases, and ROS, exacerbating inflammation, worsening demyelination, and promoting necrosis and apoptosis of damaged neurons (73) (Figure 2, upper right panel). A preliminary study find that IKK-β-dependent neutrophil activation and infiltration in the injured spinal cord aggravate neuroinflammation and neuronal damage, impairing functional recovery post-SCI (74). This phase represents the amplification of the inflammatory response, where the initial signal is dramatically magnified by the influx of potent effector cells.

### Polarization and propagation: the macrophage/microglia axis

Following the initial wave of neutrophil infiltration, monocyte-derived macrophages become the dominant immune populations at the injury site, reaching a peak around day 7 post-SCI (19) (Figure 2, lower panel). These cells originate from two sources: initially from resident microglia, and later primarily from circulating monocytes. However, due to morphological, gene expression, and functional similarities, it is challenging to distinguish between them at the injury site (75, 76). Vasandan et al. explain that macrophages are studied as key cells in postoperative regeneration due to their high plasticity. Cell therapies aiming at modulating macrophage polarization show promise for treating postoperative complications like diabetic foot (77). Macrophages are broadly classified into two main types based on surface markers, gene expression, and secreted soluble factors: M1 and M2 macrophages. These immune cells regulate tissue repair and metabolism, contributing to immune responses and anti-infection effects to maintain homeostasis (78). M1 macrophages secrete pro-inflammatory factors, like IL-6, TNF-*α*, ROS, and phospholipases, while M2 macrophages secrete anti-inflammatory factors such as IL-10 and TGF-β1 (79, 80) (Figure 2, lower panel). M1 macrophages assist in axon regeneration, whereas M2 macrophages support functional recovery. Macrophages clear apoptotic cells, which facilitate axonal regeneration and myelination (81, 82). Macrophages can shift from the M1 to the M2 phenotype in response to local microenvironment changes. This transition might potentially be achieved through local blockade of factors like scavenger receptor A or via growth factors (83). However, in spinal cord repair, such an M1-to-M2 transition is not commonly observed, and persistent M1 macrophages exacerbate secondary injury. Thus, inducing a shift toward the M2 phenotype after the acute inflammatory response may promote anatomical and functional recovery post-SCI (84, 85). Microglia, as resident CNS immune cells, also exhibit biphasic M1 (neurotoxic) and M2 (neuroprotective) polarization. They maintain neural homeostasis via synaptic pruning, clearance of abnormal proteins, and neuroprotective immune responses. The phenotypic characteristics are as follows: M1 microglia secret pro-inflammatory mediators, including IL-1β, TNF-*α*, NO, ROS; M2 microglia release anti-inflammatory factors and neurotrophins, such as IL-10, TGF-β, IGF-1 (79, 80). M1 microglia inhibit axonal regeneration and exacerbate neurotoxicity, while M2 microglia mitigate secondary damage by clearing apoptotic neuronal debris and supporting remyelination (86, 87). Although microenvironmental signals (e.g., CX3CR1/fractalkine axis or PPARγ pathway) can drive M2 polarization, in SCI pathology, the conversion from M1 to M2 is significantly impaired. Sustained M1 activation leads to amplified inflammatory cascades and progressive neuronal death. Therefore, promoting M2 polarization of microglia after the acute phase is a key strategy for optimizing the repair microenvironment (88, 89).

### Astrocyte activation and scar driving

Although astrocytes are not immune cells, they have been shown to possess molecular markers of innate and adaptive immunity post-CNS injury, holding potential for functional treatment of postoperative inflammation, such as CCL2 antibodies (e.g., Carlumab) tested in cancer (90). Studies show that locally increased IL-1β mediates the synthesis of monocyte chemoattractant protein-1 (MCP-1), keratinocyte-derived chemokine (KC), and MCP-2 via MyD88/IL-1R1 signaling in astrocytes. These chemokines may induce neutrophil and monocyte infiltration, leading to neuroinflammation at the injury site (91). Moreover, activated astrocytes post-CNS injury express and secrete various molecules, including chemokines, inflammatory cytokines, adhesion molecules, and nitric oxide, collectively forming a pro-inflammatory microenvironment. Additionally, astrocytes with inhibited NF-κB signaling significantly reduce pro-inflammatory and oxidative stress gene expression, exerting neuroprotective effects (92). Zamanian et al. find that under neuroinflammatory and ischemic conditions, astrocytes can differentiate into two subtypes: A1 and A2 astrocytes (86). The A1 phenotype significantly upregulates many genes, including those in the classical complement cascade, which are known to disrupt synaptogenesis, while the A2 phenotype promotes axonal regeneration and neuroprotection by upregulating neurotrophic factors and anti-inflammatory cytokines (87, 93). GFAP, a cytoskeletal protein in astrocytes, serves as a specific marker (88). Complement component C3 is a biomarker for A1 astrocytes but not expressed in A2 astrocytes; thus, C3/GFAP is used to identify the A1 phenotype. Since A2 astrocytes specifically express S100A10 (a member of the S100 protein family), S100A10+/GFAP serves as a dual marker for detecting the A2 phenotype (89, 94). Under the influence of IL-1β, TNF-α, and IL-17A secreted by activated M1 macrophages/microglia, neutrophils, and Th17 cells, astrocytes undergo Stat3 phosphorylation and activate into A1 astrocytes. They upregulate classical complement cascade genes (e.g., C3), pro-inflammatory factors, and chemokines, which promote neuroinflammation, synaptic loss, and secrete inhibitory molecules like CSPGs that suppress axonal regeneration and provide a molecular basis for glial scar formation (Figure 2, lower left panel).

### Chronicization and metabolic dysregulation: mitochondrial dysfunction

An imbalance between local pro- and anti-inflammatory factors serve as a hallmark of chronic inflammation in SCI (21). T lymphocytes are activated post-SCI, playing a key role in neuroinflammation and downstream cascades of neurodegeneration and repair (95). Serpe et al. find that the facial motor neuron survival after axotomy depends on anti-inflammatory CD4 + T cells, and postoperative analysis of T-cell subsets shows that both pro-inflammatory (Th1 and Th17) and anti-inflammatory (Th2 and Treg) T cells are activated after injury (96). Disruption of the balance between Th1/Th2 and Th17/Treg cells skews the adaptive immune response towards the pro-inflammatory Th1 and Th17 phenotypes, increasing release of pro-inflammatory cytokines such as IFN-*γ*, TNF-*β*, and IL-17 (97). Additionally, these cells promote the synthesis and release of autoantibodies by B lymphocytes, which further cause neuronal demyelination and axonal damage (95). Research demonstrates that miR-155 deficiency significantly inhibits the Th17 differentiation of CD4 + T cells post-SCI and promotes functional recovery by suppressing IL-17 expression (98). Therefore, inducing a shift towards the Th2 and Treg phenotypes may be neuroprotective in the early postoperative stage of SCI. Chronic inflammation and oxidative stress form a vicious cycle, leading to excessive ROS production by mitochondria (99). Increased ROS directly damage neuronal membrane lipids and activate the ASK1-JNK pathway or inhibit the NRF2 antioxidant pathway, downregulating stress-induced heme oxygenase-1 (HO-1) and superoxide dismutase 2 (SOD2), triggering oligodendrocyte apoptosis (100, 101). Reduced activity of the Parkin/PINK1 pathway post-SCI leads to the accumulation of damaged mitochondria, causing ROS and mtDNA release into the cytoplasm, activating the cGAS-STING pathway and secreting type I interferon (IFN-β), amplifying inflammation (102, 103). Preclinical studies demonstrate that antioxidants like edaravone and N-acetylcysteine can reduce spinal ROS levels, increase axonal survival, and improve motor function scores (104–106). Recent studies also note the occurrence of ferroptosis during chronic inflammation. Accumulated lipid peroxidation products (e.g., MDA, 4-HNE) post-SCI inhibit GPX4 activity, oxidizing membrane polyunsaturated fatty acids (PUFAs) and inducing iron-dependent cell death (107, 108). Inhibiting key enzymes in lipid metabolism, such as acyl-CoA synthetase long-chain family member 4 (ACSL4), can reduce ferroptosis and increase neuronal survival (109).

## Interaction of the glial scar–inflammation axis

As highlighted in Figure 1, the mature glial scar is not merely a passive barrier but a dynamic structure composed of reaction astrocytes, activated fibroblasts, infiltrating monocytes, and a dense network of CSPGs and ECM. While this scar serves to contain the injury and prevent spread of inflammation, its primary consequence is the creation of a formidable obstacle to axonal sprouting. Axons attempting to regenerate encounter this inhibitory environment and are effectively blocked, preventing functional neural circuit reorganization and recovery.

### Inflammation-driven glial scar formation

In the postoperative period of SCI in CSM, excessive activation of the inflammatory response serves as a primary cause of neuroglial scar formation (21). Post-SCI, neutrophils and inflammatory macrophages rapidly infiltrate the injury site, producing large quantities of inflammatory cytokines (e.g., IL-1β, TNF-*α*), triggering signaling pathways in astrocytes and microglia and driving their transformation (38, 110). Inflammatory cytokines can directly act on astrocytes, promoting their proliferation and the secretion of ECM components like CSPGs, which provides the molecular basis for scar formation. IL-17 produced by Th17 cells stimulates glial activity, promoting inflammation and glial scar formation (111, 112). Clinical observations show that patients with persistently high inflammatory factors often exhibit more severe scar hyperplasia (113), and the inflammatory microenvironment may synergistically promote glial scar formation through various mechanisms (36).

### Bidirectional regulation of inflammation by glial scar

During the process of injury repair, the glial scar plays a dynamic and bidirectional regulatory role. Initially, the scar functions as a physical barrier to prevent inflammation spread and temporarily protects normal tissue by secreting proteins that inhibit destructive enzymes (21, 36). However, as the glial scar matures, its composition changes, incorporating components that promote inflammation (37). Dense ECM can inhibit the clearance of immune cells from the injury area, and, via the activation of relevant receptor pathways, it polarizes microglia and macrophages toward pro-inflammatory phenotypes, leading to an increased secretion of inflammatory mediators (21, 114). Additionally, changes in the mechanical properties of the glial scar can activate mechanosensitive signaling, attracting peripheral immune cell infiltration and inducing abnormal adaptive immune responses (115). This shift from protective to detrimental effects provides a structural basis for persistent inflammation in the chronic phase, representing a spatiotemporally interconnected and bidirectional regulatory pattern.

### Vicious cycle of mutual amplification

Interactions between the neuroglial scar and inflammation ultimately form a self-reinforcing pathological cycle. Deposits in the scar matrix continuously trap/release DAMPs, maintaining perpetual innate immune signaling and recurrent generation of inflammatory factors. These factors, in turn, promote scar matrix synthesis and stiffening (116, 117). Chronic inflammation leads to an excess of reactive ROS and metabolic disorders, disrupting mitochondrial function and impairing cellular self-repair (118). Furthermore, the physical barrier of the scar impedes the transport of neurotrophic factors (119). This vicious cycle results in a gradual imbalance in the postoperative spinal microenvironment, sustained chronic inflammation, continuous scar expansion, inhibited axonal regeneration and myelination, and ultimately hindered neurological recovery or even degeneration. Clinically, this mutual amplification mechanism may underlie postoperative recurrence or symptom exacerbation in some patients (21, 116, 119, 120).

## Therapeutic approaches

Current treatments for neuroinflammation post-SCI are extremely limited. Methylprednisolone sodium succinate stands as the only FDA-approved drug for clinical use. It primarily exerts its effects by binding to glucocorticoid receptors and preventing pro-inflammatory transcription factors from entering the nucleus (121). However, due to its heterogeneous therapeutic effects and serious side effects, such as gastrointestinal bleeding, femoral head necrosis, and wound infection, its clinical application is limited (122). In recent years, regenerative medicine has made significant advances in SCI treatment. Stem cell therapy, which employs cell replacement, neurotrophic factor secretion, and modulation of the extracellular microenvironment to repair damaged neural tissue, has emerged as a promising treatment for post-CSM injury (123). Studies worldwide have isolated various pluripotent stem cells from human tissues and organs, including human embryonic stem cells (hESCs), neural stem cells (NSCs), human umbilical cord blood stem cells (UHSCs), placenta-derived stem cells (PNSCs), and bone marrow stromal cells (MSCs) (124, 125).

Pluripotent stem cells can significantly improve various functional parameters in SCI models. Although embryonic stem cells possess multi-differentiation potential, ethical concerns and tumorigenic risks have shifted focus toward adult stem cells (126). Umbilical cord blood stem cells are easily accessible and exhibit low immunogenicity, thus garnering significant attention. Animal models confirm that these cells inhibit the expression of apoptotic genes, such as Fas and caspase, reduce demyelination, and promote axonal regeneration via the secretion of neurotrophic factors (127). In addition, bone marrow MSCs can reach the injury site via intravenous or local transplantation. They differentiate into Schwann cells, form myelin, and release VEGF and BDNF, thereby promoting local microcirculation (128, 129). Primate studies demonstrate that transplanted neural progenitor cells can differentiate into neurons and oligodendrocytes, significantly improving forelimb grasping function. These findings provide important experimental basis for clinical translation (130).

Current research reveals that MSCs primarily exert therapeutic effects by mediating intercellular interactions or secreting cytokines to achieve anti-inflammatory outcomes (131). Since glial scars are mainly caused by astrocyte activation, reducing astrocyte activation may be an effective way to diminish scarring. Studies have confirmed that triptolide, rolipram, HDAC3 inhibitors, astragaloside IV, and intravenous immunoglobulin can reduce glial scar formation by inhibiting astrocyte activation (124, 132–135). In summary, the main challenge remains poor neurological recovery post-CSM surgery. The neuroglial scar initially inhibits inflammation spread but later becomes a regenerative barrier in the chronic phase. Issues such as *in vitro* differentiation induction post-stem cell transplantation, *in vivo* targeted drug delivery to stem cells and carriers, and synergistic effects of combination therapy require further study (122). Moreover, immune rejection of allogeneic stem cells, the safety of gene editing, and the biocompatibility of 3D scaffolds also need to be addressed (136). Future exploration should have focused on developing controllable functional materials (e.g., pH- or enzyme-responsive), single-cell level microenvironment modulation technologies, and precision therapy based on targeted immune molecules.

## Summary and prospects

With the aging of the population, the incidence and prevalence of CSM will continue to increase. Surgical decompression, the main treatment for alleviating CSM symptoms, may still worsen symptoms or lead to disease recurrence postoperatively. Post-CSM secondary injury involves multiple immune cells and molecular reactions, where inflammation and glial scar formation are major obstacles to neuronal anatomical and functional repair, determining disease progression and prognosis. Consequently, they have become research hotspots in post-CSM repair. Various immune factors influencing spinal secondary injury and targeted treatments have been proposed. This review introduces the “glial scar–inflammation axis,” shifting the perspective from “structure–function” to “immune microenvironment” to elucidate molecular mechanisms of post-CSM recurrence.

However, a critical appraisal of the current literature reveals a substantial disconnect between preclinical mechanistic understanding and clinical validation. The overwhelming majority of evidence supporting the glial scar–inflammation axis derives from animal models of acute traumatic SCI, which differ fundamentally from the chronic, progressive compression pathology characteristic of CSM. Direct clinical evidence specifically examining this axis in postoperative CSM patients is remarkably sparse, consisting primarily of indirect cytokine correlations and non-specific imaging findings. This evidence gap represents the single most important limitation in the field and must be addressed before the glial scar–inflammation axis can be considered a validated therapeutic target in CSM.

Given the dual roles of neuroglial scars and inflammation in the postoperative microenvironment, new therapeutic strategies should amplify and enhance their reparative effects while suppressing inflammatory responses. Future research must prioritize translational studies that bridge this evidence gap. Key priorities include: (1) establishing human tissue biobanks from CSM patients to enable histopathological characterization of glial scar formation; (2) developing and validating non-invasive imaging biomarkers capable of quantifying glial scar burden and neuroinflammation in living patients; (3) conducting prospective cohort studies linking perioperative inflammatory and scarring biomarkers to long-term clinical outcomes and recurrence; and (4) designing in-human clinical trials of targeted immunomodulatory or anti-scarring therapies specifically for CSM populations. Future clinical innovations may involve signal pathway inhibitors, targeted drug therapies, mesenchymal stem cell treatments, and biodegradable materials to minimize surgical trauma and reduce initial inflammation activation (27).

In conclusion, the glial scar–inflammation axis plays a crucial role in post-CSM repair. Key factors in preventing recurrence or symptom exacerbation may lie within the myriad immune interactions involved in SCI or neurodegenerative diseases. Combined therapies targeting neuroglial scar–inflammation interactions require further exploration, and it is believed that this field will yield many more new research findings.

## References

[1] Emery SE Bohlman HH Bolesta MJ Jones PK. Anterior cervical decompression and arthrodesis for the treatment of cervical spondylotic myelopathy. Two to seventeen-year follow-up. J Bone Joint Surg Am. (1998) 80:941–51. doi: 10.2106/00004623-199807000-00002, 9697998

[2] Badhiwala JH Ahuja CS Akbar MA Witiw CD Nassiri F Furlan JC . Degenerative cervical myelopathy - update and future directions. Nat Rev Neurol. (2020) 16:108–24. doi: 10.1038/s41582-019-0303-0, 31974455

[3] Klineberg E. Cervical spondylotic myelopathy: a review of the evidence. Orthop Clin North Am. (2010) 41:193–202. doi: 10.1016/j.ocl.2009.12.010 20399358

[4] Iyer A Azad TD Tharin S. Cervical Spondylotic myelopathy. Clinical Spine Surgery. (2016) 29:408–14. doi: 10.1097/bsd.0000000000000397 27352369

[5] Tetreault L Ibrahim A Côté P Singh A Fehlings MG. A systematic review of clinical and surgical predictors of complications following surgery for degenerative cervical myelopathy. J Neurosurg Spine. (2016) 24:77–99. doi: 10.3171/2015.3.SPINE14971, 26407090

[6] Kim PK Alexander JT. Indications for circumferential surgery for cervical spondylotic myelopathy. Spine J. (2006) 6:S299–307. doi: 10.1016/j.spinee.2006.04.025, 17097550

[7] Ramesh VG Kannan MGV Sriram K Balasubramanian C. Prognostication in cervical spondylotic myelopathy: proposal for a new simple practical scoring system. Asian J Neurosurg. (2017) 12:525–8. doi: 10.4103/1793-5482.146391, 28761535 PMC5532942

[8] Sadasivan KK Reddy RP Albright JA. The natural history of cervical spondylotic myelopathy. Yale J Biol Med. (1993) 66:235–42. 8209559 PMC2588865

[9] Ghogawala Z Terrin N Dunbar MR Breeze JL Freund KM Kanter AS . Effect of ventral vs dorsal spinal surgery on patient-reported physical functioning in patients with cervical Spondylotic myelopathy: a randomized clinical trial. JAMA. (2021) 325:942–51. doi: 10.1001/jama.2021.1233, 33687463 PMC7944378

[10] Li H Dai LY. A systematic review of complications in cervical spine surgery for ossification of the posterior longitudinal ligament. Spine J. (2011) 11:1049–57. doi: 10.1016/j.spinee.2011.09.008, 22015235

[11] Chagas H Domingues F Aversa A Fonseca ALV de Souza JM. Cervical spondylotic myelopathy: 10 years of prospective outcome analysis of anterior decompression and fusion. Surg Neurol. (2005) 64:30–5. doi: 10.1016/j.surneu.2005.02.016 15967227

[12] Sakaguchi T Heyder A Tanaka M Uotani K Omori T Kodama Y . Rehabilitation to improve outcomes after cervical spine surgery: narrative review. J Clin Med. (2024) 13:5363. doi: 10.3390/jcm13185363, 39336849 PMC11432758

[13] Shin JJ Jin BH Kim KS Cho YE Cho WH. Intramedullary high signal intensity and neurological status as prognostic factors in cervical spondylotic myelopathy. Acta Neurochir. (2010) 152:1687–94. doi: 10.1007/s00701-010-0692-8, 20512384

[14] Shin JW Jin SW Kim SH Choi JI Kim BJ Kim SD . Predictors of outcome in patients with cervical Spondylotic myelopathy undergoing unilateral open-door Laminoplasty. Korean J Spine. (2015) 12:261–6. doi: 10.14245/kjs.2015.12.4.261, 26834814 PMC4731561

[15] Bakhsheshian J Mehta VA Liu JC. Current diagnosis and Management of Cervical Spondylotic Myelopathy. Global Spine J. (2017) 7:572–86. doi: 10.1177/2192568217699208, 28894688 PMC5582708

[16] Clifford T Finkel Z Rodriguez B Joseph A Cai L. Current advancements in spinal cord injury research-glial scar formation and neural regeneration. Cells. (2023) 12:853. doi: 10.3390/cells12060853, 36980193 PMC10046908

[17] Donnally CJ 3rd Patel PD Canseco JA Vaccaro AR Kepler CK. Current Management of Cervical Spondylotic Myelopathy. Clin Spine Surg. (2022) 35:E68–e76. doi: 10.1097/BSD.0000000000001113, 34379614

[18] Fan B Wei Z Yao X Shi G Cheng X Zhou X . Microenvironment imbalance of spinal cord injury. Cell Transplant. (2018) 27:853–66. doi: 10.1177/0963689718755778, 29871522 PMC6050904

[19] Milich LM Ryan CB Lee JK. The origin, fate, and contribution of macrophages to spinal cord injury pathology. Acta Neuropathol. (2019) 137:785–97. doi: 10.1007/s00401-019-01992-3, 30929040 PMC6510275

[20] Milich LM Choi JS Ryan C Cerqueira SR Benavides S Yahn SL . Single-cell analysis of the cellular heterogeneity and interactions in the injured mouse spinal cord. J Exp Med. (2021) 218:e20210040. doi: 10.1084/jem.20210040, 34132743 PMC8212781

[21] David S Kroner A. Repertoire of microglial and macrophage responses after spinal cord injury. Nat Rev Neurosci. (2011) 12:388–99. doi: 10.1038/nrn3053, 21673720

[22] Sofroniew MV. Dissecting spinal cord regeneration. Nature. (2018) 557:343–50. doi: 10.1038/s41586-018-0068-4, 29769671

[23] Gensel JC Zhang B. Macrophage activation and its role in repair and pathology after spinal cord injury. Brain Res. (2015) 1619:1–11. doi: 10.1016/j.brainres.2014.12.045, 25578260

[24] Michelucci A Mittelbronn M Gomez-Nicola D. Microglia in health and disease: a unique immune cell population. Front Immunol. (2018) 9:1779. doi: 10.3389/fimmu.2018.01779, 30158924 PMC6104119

[25] Choudhary OP. Animal models for surgeries and implants: a vital tool in medical research and development. Annals Medicine Surgery. (2025) 87:4090–5. doi: 10.1097/MS9.0000000000003400, 40851997 PMC12369812

[26] Choudhary OP Sarkar R. Animal anatomical teaching models for enhanced veterinary anatomy education and learning. Pak Vet J. (2025) 45:961–82. doi: 10.29261/pakvetj/2025.250

[27] Bradbury EJ Moon LD Popat RJ King VR Bennett GS Patel PN . Chondroitinase ABC promotes functional recovery after spinal cord injury. Nature. (2002) 416:636–40. doi: 10.1038/416636a, 11948352

[28] Wang W Ren Y Xu F Zhang X Wang F Wang T . Identification of hub genes significantly linked to temporal lobe epilepsy and apoptosis via bioinformatics analysis. Front Mol Neurosci. (2024) 17:1300348. doi: 10.3389/fnmol.2024.1300348, 38384278 PMC10879302

[29] Tran AP Warren PM Silver J. The biology of regeneration failure and success after spinal cord injury. Physiol Rev. (2018) 98:881–917. doi: 10.1152/physrev.00017.2017, 29513146 PMC5966716

[30] Karadimas SK Gialeli CH Klironomos G Tzanakakis GN Panagiotopoulos E Karamanos NK . The role of oligodendrocytes in the molecular pathobiology and potential molecular treatment of cervical spondylotic myelopathy. Curr Med Chem. (2010) 17:1048–58. doi: 10.2174/092986710790820598, 20156160

[31] Oyinbo CA. Secondary injury mechanisms in traumatic spinal cord injury: a nugget of this multiply cascade. Acta Neurobiol Exp. (2011) 71:281–99. doi: 10.55782/ane-2011-1848, 21731081

[32] Li GS Chen GH Wang KH Wang XX Hu XS Wei B . Neurovascular unit compensation from adjacent level may contribute to spontaneous functional recovery in experimental cervical spondylotic myelopathy. Int J Mol Sci. (2023) 24:3408. doi: 10.3390/ijms24043408, 36834841 PMC9962900

[33] Klune JR Dhupar R Cardinal J Billiar TR Tsung A. HMGB1: endogenous danger signaling. Mol Med. (2008) 14:476–84. doi: 10.2119/2008-00034.Klune, 18431461 PMC2323334

[34] Davalos D Grutzendler J Yang G Kim JV Zuo Y Jung S . ATP mediates rapid microglial response to local brain injury in vivo. Nat Neurosci. (2005) 8:752–8. doi: 10.1038/nn1472, 15895084

[35] Fawcett JW Asher RA. The glial scar and central nervous system repair. Brain Res Bull. (1999) 49:377–91. doi: 10.1016/s0361-9230(99)00072-6, 10483914

[36] Silver J Miller JH. Regeneration beyond the glial scar. Nat Rev Neurosci. (2004) 5:146–56. doi: 10.1038/nrn1326, 14735117

[37] Anderson MA Burda JE Ren Y Ao Y O'Shea TM Kawaguchi R . Astrocyte scar formation aids central nervous system axon regeneration. Nature. (2016) 532:195–200. doi: 10.1038/nature17623, 27027288 PMC5243141

[38] Sofroniew MV. Molecular dissection of reactive astrogliosis and glial scar formation. Trends Neurosci. (2009) 32:638–47. doi: 10.1016/j.tins.2009.08.002, 19782411 PMC2787735

[39] Pekny M Pekna M. Astrocyte intermediate filaments in CNS pathologies and regeneration. J Pathol. (2004) 204:428–37. doi: 10.1002/path.1645, 15495269

[40] Wilhelmsson U Li L Pekna M Berthold CH Blom S Eliasson C . Absence of glial fibrillary acidic protein and vimentin prevents hypertrophy of astrocytic processes and improves post-traumatic regeneration. J Neurosci. (2004) 24:5016–21. doi: 10.1523/JNEUROSCI.0820-04.2004, 15163694 PMC6729371

[41] Wang L Wang H Zhu M Ni X Sun L Wang W . Platelet-derived TGF-β1 induces functional reprogramming of myeloid-derived suppressor cells in immune thrombocytopenia. Blood. (2024) 144:99–112. doi: 10.1182/blood.2023022738, 38574321

[42] Hellal F Hurtado A Ruschel J Flynn KC Laskowski CJ Umlauf M . Microtubule stabilization reduces scarring and causes axon regeneration after spinal cord injury. Science. (2011) 331:928–31. doi: 10.1126/science.1201148, 21273450 PMC3330754

[43] Shen Y Tenney AP Busch SA Horn KP Cuascut FX Liu K . PTPsigma is a receptor for chondroitin sulfate proteoglycan, an inhibitor of neural regeneration. Science. (2009) 326:592–6. doi: 10.1126/science.1178310, 19833921 PMC2811318

[44] Yong VW Power C Forsyth P Edwards DR. Metalloproteinases in biology and pathology of the nervous system. Nat Rev Neurosci. (2001) 2:502–11. doi: 10.1038/35081571, 11433375 PMC7097548

[45] Moeendarbary E Weber IP Sheridan GK Koser DE Soleman S Haenzi B . The soft mechanical signature of glial scars in the central nervous system. Nat Commun. (2017) 8:14787. doi: 10.1038/ncomms14787, 28317912 PMC5364386

[46] Anjum A Yazid MD Fauzi Daud M Idris J Ng AMH Selvi Naicker A . Spinal cord injury: pathophysiology, multimolecular interactions, and underlying recovery mechanisms. Int J Mol Sci. (2020) 21:7533. doi: 10.3390/ijms21207533 33066029 PMC7589539

[47] Zeng H Liu N Yang YY Xing HY Liu XX Li F . Lentivirus-mediated downregulation of α-synuclein reduces neuroinflammation and promotes functional recovery in rats with spinal cord injury. J Neuroinflammation. (2019) 16:283. doi: 10.1186/s12974-019-1658-2, 31888724 PMC6936070

[48] Faulkner JR Herrmann JE Woo MJ Tansey KE Doan NB Sofroniew MV. Reactive astrocytes protect tissue and preserve function after spinal cord injury. J Neurosci. (2004) 24:2143–55. doi: 10.1523/JNEUROSCI.3547-03.2004, 14999065 PMC6730429

[49] Whiting AC Turner JD. Astrocytic scar facilitates axon regeneration after spinal cord injury. World Neurosurg. (2016) 96:591–2. doi: 10.1016/j.wneu.2016.10.050, 27746254

[50] Anderson MA O'Shea TM Burda JE Ao Y Barlatey SL Bernstein AM . Required growth facilitators propel axon regeneration across complete spinal cord injury. Nature. (2018) 561:396–400. doi: 10.1038/s41586-018-0467-6, 30158698 PMC6151128

[51] Dias DO Göritz C. Fibrotic scarring following lesions to the central nervous system. Matrix Biol. (2018) 68-69:561–70. doi: 10.1016/j.matbio.2018.02.009, 29428230

[52] Siebert JR Osterhout DJ. Select neurotrophins promote oligodendrocyte progenitor cell process outgrowth in the presence of chondroitin sulfate proteoglycans. J Neurosci Res. (2021) 99:1009–23. doi: 10.1002/jnr.24780, 33453083 PMC7986866

[53] Mukherjee N Nandi S Garg S Ghosh S Ghosh S Samat R . Targeting chondroitin sulfate proteoglycans: an emerging therapeutic strategy to treat CNS injury. ACS Chem Neurosci. (2020) 11:231–2. doi: 10.1021/acschemneuro.0c00004, 31939650

[54] Tran AP Warren PM Silver J. New insights into glial scar formation after spinal cord injury. Cell Tissue Res. (2022) 387:319–36. doi: 10.1007/s00441-021-03477-w, 34076775 PMC8975767

[55] Dyck SM Karimi-Abdolrezaee S. Chondroitin sulfate proteoglycans: key modulators in the developing and pathologic central nervous system. Exp Neurol. (2015) 269:169–87. doi: 10.1016/j.expneurol.2015.04.006, 25900055

[56] Takeuchi K Yoshioka N Higa Onaga S Watanabe Y Miyata S Wada Y . Chondroitin sulphate N-acetylgalactosaminyl-transferase-1 inhibits recovery from neural injury. Nat Commun. (2013) 4:2740. doi: 10.1038/ncomms3740, 24220492 PMC3831297

[57] Hussein RK Mencio CP Katagiri Y Brake AM Geller HM. Role of chondroitin sulfation following spinal cord injury. Front Cell Neurosci. (2020) 14:208. doi: 10.3389/fncel.2020.00208, 32848612 PMC7419623

[58] Lang BT Cregg JM DePaul MA Tran AP Xu K Dyck SM . Modulation of the proteoglycan receptor PTPσ promotes recovery after spinal cord injury. Nature. (2015) 518:404–8. doi: 10.1038/nature13974, 25470046 PMC4336236

[59] Stipursky J Gomes FC. TGF-beta1/SMAD signaling induces astrocyte fate commitment in vitro: implications for radial glia development. Glia. (2007) 55:1023–33. doi: 10.1002/glia.20522, 17549683

[60] Susarla BT Laing ED Yu P Katagiri Y Geller HM Symes AJ. Smad proteins differentially regulate transforming growth factor-β-mediated induction of chondroitin sulfate proteoglycans. J Neurochem. (2011) 119:868–78. doi: 10.1111/j.1471-4159.2011.07470.x, 21895657 PMC3197872

[61] Shen D Wang X Gu X. Scar-modulating treatments for central nervous system injury. Neurosci Bull. (2014) 30:967–84. doi: 10.1007/s12264-013-1456-2, 24957881 PMC5562555

[62] Yao XQ Liu ZY Chen JY Huang ZC Liu JH Sun BH . Proteomics and bioinformatics reveal insights into neuroinflammation in the acute to subacute phases in rat models of spinal cord contusion injury. FASEB J. (2021) 35:e21735. doi: 10.1096/fj.202100081RR, 34143440

[63] Maldonado-Lasunción I Verhaagen J Oudega M. Mesenchymal stem cell-macrophage choreography supporting spinal cord repair. Neurotherapeutics. (2018) 15:578–87. doi: 10.1007/s13311-018-0629-0, 29728851 PMC6095786

[64] Dokalis N Prinz M. Resolution of neuroinflammation: mechanisms and potential therapeutic option. Semin Immunopathol. (2019) 41:699–709. doi: 10.1007/s00281-019-00764-1, 31705317

[65] Dumont CM Margul DJ Shea LD. Tissue engineering approaches to modulate the inflammatory milieu following spinal cord injury. Cells Tissues Organs. (2016) 202:52–66. doi: 10.1159/000446646, 27701152 PMC5067186

[66] Chio JCT Xu KJ Popovich P David S Fehlings MG. Neuroimmunological therapies for treating spinal cord injury: evidence and future perspectives. Exp Neurol. (2021) 341:113704. doi: 10.1016/j.expneurol.2021.113704, 33745920

[67] Kigerl KA Gensel JC Ankeny DP Alexander JK Donnelly DJ Popovich PG. Identification of two distinct macrophage subsets with divergent effects causing either neurotoxicity or regeneration in the injured mouse spinal cord. J Neurosci. (2009) 29:13435–44. doi: 10.1523/JNEUROSCI.3257-09.2009, 19864556 PMC2788152

[68] Garcia E Aguilar-Cevallos J Silva-Garcia R Ibarra A. Cytokine and growth factor activation in vivo and in vitro after spinal cord injury. Mediat Inflamm 2016(9476020. (2016) 2016:1–21. doi: 10.1155/2016/9476020, 27418745 PMC4935915

[69] Kigerl KA de Rivero Vaccari JP Dietrich WD Popovich PG Keane RW. Pattern recognition receptors and central nervous system repair. Exp Neurol. (2014) 258:5–16. doi: 10.1016/j.expneurol.2014.01.001, 25017883 PMC4974939

[70] Paramos-de-Carvalho D Martins I Cristóvão AM Dias AF Neves-Silva D Pereira T . Targeting senescent cells improves functional recovery after spinal cord injury. Cell Rep. (2021) 36:109334. doi: 10.1016/j.celrep.2021.109334, 34233184

[71] Neirinckx V Coste C Franzen R Gothot A Rogister B Wislet S. Neutrophil contribution to spinal cord injury and repair. J Neuroinflammation. (2014) 11:150. doi: 10.1186/s12974-014-0150-2, 25163400 PMC4174328

[72] Yokota K Saito T Kobayakawa K Kubota K Hara M Murata M . The feasibility of in vivo imaging of infiltrating blood cells for predicting the functional prognosis after spinal cord injury. Sci Rep. (2016) 6:25673. doi: 10.1038/srep25673, 27156468 PMC4860707

[73] Kubota K Saiwai H Kumamaru H Maeda T Ohkawa Y Aratani Y . Myeloperoxidase exacerbates secondary injury by generating highly reactive oxygen species and mediating neutrophil recruitment in experimental spinal cord injury. Spine. (2012) 37:1363–9. doi: 10.1097/BRS.0b013e31824b9e77, 22322369

[74] Kang J Jiang MH Min HJ Jo EK Lee S Karin M . IKK-β-mediated myeloid cell activation exacerbates inflammation and inhibits recovery after spinal cord injury. Eur J Immunol. (2011) 41:1266–77. doi: 10.1002/eji.201040582 21469085

[75] Devanney NA Stewart AN Gensel JC. Microglia and macrophage metabolism in CNS injury and disease: the role of immunometabolism in neurodegeneration and neurotrauma. Exp Neurol. (2020) 329:113310. doi: 10.1016/j.expneurol.2020.113310, 32289316 PMC7237336

[76] David S Greenhalgh AD Kroner A. Macrophage and microglial plasticity in the injured spinal cord. Neuroscience. (2015) 307:311–8. doi: 10.1016/j.neuroscience.2015.08.064, 26342747

[77] Vasandan AB Jahnavi S Shashank C Prasad P Kumar A Prasanna SJ. Human mesenchymal stem cells program macrophage plasticity by altering their metabolic status via a PGE(2)-dependent mechanism. Sci Rep. (2016) 6:38308. doi: 10.1038/srep38308, 27910911 PMC5133610

[78] Norden DM Faw TD McKim DB Deibert RJ Fisher LC Sheridan JF . Bone marrow-derived monocytes drive the inflammatory microenvironment in local and remote regions after thoracic spinal cord injury. J Neurotrauma. (2019) 36:937–49. doi: 10.1089/neu.2018.5806, 30014767 PMC6484351

[79] Sica A Mantovani A. Macrophage plasticity and polarization: in vivo veritas. J Clin Invest. (2012) 122:787–95. doi: 10.1172/JCI59643, 22378047 PMC3287223

[80] Kobashi S Terashima T Katagi M Nakae Y Okano J Suzuki Y . Transplantation of M2-deviated microglia promotes recovery of motor function after spinal cord injury in mice. Molecular Ther. (2020) 28:254–65. doi: 10.1016/j.ymthe.2019.09.004, 31604678 PMC6952178

[81] Han GH Kim SJ Ko WK Lee D Han IB Sheen SH . Transplantation of tauroursodeoxycholic acid-inducing M2-phenotype macrophages promotes an anti-neuroinflammatory effect and functional recovery after spinal cord injury in rats. Cell Prolif. (2021) 54:e13050. doi: 10.1111/cpr.13050, 33960559 PMC8168422

[82] Zhou X He X Ren Y. Function of microglia and macrophages in secondary damage after spinal cord injury. Neural Regen Res. (2014) 9:1787–95. doi: 10.4103/1673-5374.143423, 25422640 PMC4239768

[83] Chazaud B. Inflammation and skeletal muscle regeneration: leave it to the macrophages! Trends Immunol. (2020) 41:481–92. doi: 10.1016/j.it.2020.04.006, 32362490

[84] Funes SC Rios M Escobar-Vera J Kalergis AM. Implications of macrophage polarization in autoimmunity. Immunology. (2018) 154:186–95. doi: 10.1111/imm.12910, 29455468 PMC5980179

[85] Liu W Tang P Wang J Ye W Ge X Rong Y . Extracellular vesicles derived from melatonin-preconditioned mesenchymal stem cells containing USP29 repair traumatic spinal cord injury by stabilizing NRF2. J Pineal Res. (2021) 71:e12769. doi: 10.1111/jpi.12769, 34562326

[86] Zamanian JL Xu L Foo LC Nouri N Zhou L Giffard RG . Genomic analysis of reactive astrogliosis. J Neurosci. (2012) 32:6391–410. doi: 10.1523/jneurosci.6221-11.2012, 22553043 PMC3480225

[87] Liddelow SA Barres BA. Reactive astrocytes: production, function, and therapeutic potential. Immunity. (2017) 46:957–67. doi: 10.1016/j.immuni.2017.06.006, 28636962

[88] Nagao M Ogata T Sawada Y Gotoh Y. Zbtb20 promotes astrocytogenesis during neocortical development. Nat Commun. (2016) 7:11102. doi: 10.1038/ncomms11102, 27000654 PMC4804180

[89] Hassanzadeh S Jalessi M Jameie SB Khanmohammadi M Bagher Z Namjoo Z . More attention on glial cells to have better recovery after spinal cord injury. Biochem Biophys Rep. (2021) 25:100905. doi: 10.1016/j.bbrep.2020.100905, 33553683 PMC7844125

[90] Colombo E Farina C. Astrocytes: key regulators of neuroinflammation. Trends Immunol. (2016) 37:608–20. doi: 10.1016/j.it.2016.06.006, 27443914

[91] Pineau I Sun L Bastien D Lacroix S. Astrocytes initiate inflammation in the injured mouse spinal cord by promoting the entry of neutrophils and inflammatory monocytes in an IL-1 receptor/MyD88-dependent fashion. Brain Behav Immun. (2010) 24:540–53. doi: 10.1016/j.bbi.2009.11.007, 19932745

[92] Dvoriantchikova G Barakat D Brambilla R Agudelo C Hernandez E Bethea JR . Inactivation of astroglial NF-kappa B promotes survival of retinal neurons following ischemic injury. Eur J Neurosci. (2009) 30:175–85. doi: 10.1111/j.1460-9568.2009.06814.x, 19614983 PMC2778328

[93] Liddelow SA Guttenplan KA Clarke LE Bennett FC Bohlen CJ Schirmer L . Neurotoxic reactive astrocytes are induced by activated microglia. Nature. (2017) 541:481–7. doi: 10.1038/nature21029, 28099414 PMC5404890

[94] Li X Li M Tian L Chen J Liu R Ning B. Reactive astrogliosis: implications in spinal cord injury progression and therapy. Oxidative Med Cell Longev. (2020) 2020:9494352. doi: 10.1155/2020/9494352, 32884625 PMC7455824

[95] Ankeny DP Popovich PG. Mechanisms and implications of adaptive immune responses after traumatic spinal cord injury. Neuroscience. (2009) 158:1112–21. doi: 10.1016/j.neuroscience.2008.07.001, 18674593 PMC2661571

[96] Serpe CJ Coers S Sanders VM Jones KJ. CD4+ T, but not CD8+ or B, lymphocytes mediate facial motoneuron survival after facial nerve transection. Brain Behav Immun. (2003) 17:393–402. doi: 10.1016/s0889-1591(03)00028-x, 12946661

[97] Moalem G Gdalyahu A Shani Y Otten U Lazarovici P Cohen IR . Production of neurotrophins by activated T cells: implications for neuroprotective autoimmunity. J Autoimmun. (2000) 15:331–45. doi: 10.1006/jaut.2000.0441, 11040074

[98] Yi J Wang D Niu X Hu J Zhou Y Li Z. MicroRNA-155 deficiency suppresses Th17 cell differentiation and improves locomotor recovery after spinal cord injury. Scand J Immunol. (2015) 81:284–90. doi: 10.1111/sji.12276, 25651871

[99] West AP Khoury-Hanold W Staron M Tal MC Pineda CM Lang SM . Mitochondrial DNA stress primes the antiviral innate immune response. Nature. (2015) 520:553–7. doi: 10.1038/nature14156, 25642965 PMC4409480

[100] Vargas MR Johnson DA Sirkis DW Messing A Johnson JA. Nrf2 activation in astrocytes protects against neurodegeneration in mouse models of familial amyotrophic lateral sclerosis. J Neurosci. (2008) 28:13574–81. doi: 10.1523/JNEUROSCI.4099-08.2008, 19074031 PMC2866507

[101] Yang Y Liu Y Zhang Y Ji W Wang L Lee SC. Periplogenin activates ROS-ER stress pathway to trigger apoptosis via BIP-eIF2α- CHOP and IRE1α-ASK1-JNK signaling routes. Anti Cancer Agents Med Chem. (2021) 21:61–70. doi: 10.2174/1871520620666200708104559, 32640963

[102] Lee JJ Andreazza S Whitworth AJ. The STING pathway does not contribute to behavioural or mitochondrial phenotypes in Drosophila Pink1/parkin or mtDNA mutator models. Sci Rep. (2020) 10:2693. doi: 10.1038/s41598-020-59647-3, 32060339 PMC7021792

[103] Pickrell AM Youle RJ. The roles of PINK1, parkin, and mitochondrial fidelity in Parkinson's disease. Neuron. (2015) 85:257–73. doi: 10.1016/j.neuron.2014.12.007, 25611507 PMC4764997

[104] Fu D Liu H Liu H Yao J. Effects of D-Ala2, D-Leu5-Enkephalin pre- and post-conditioning in a rabbit model of spinal cord ischemia and reperfusion injury. Mol Med Rep. (2019) 20:4811–20. doi: 10.3892/mmr.2019.10729, 31638217 PMC6854538

[105] Michaličková D Öztürk HK Hroudová J Ľupták M Kučera T Hrnčíř T . Edaravone attenuates disease severity of experimental auto-immune encephalomyelitis and increases gene expression of Nrf2 and HO-1. Physiol Res. (2022) 71:147–57. doi: 10.33549/physiolres.934800, 35043649 PMC8997675

[106] Guo J Li Y Chen Z He Z Zhang B Li Y . N-acetylcysteine treatment following spinal cord trauma reduces neural tissue damage and improves locomotor function in mice. Mol Med Rep. (2015) 12:37–44. doi: 10.3892/mmr.2015.3390, 25738883 PMC4438879

[107] Yang WS SriRamaratnam R Welsch ME Shimada K Skouta R Viswanathan VS . Regulation of ferroptotic cancer cell death by GPX4. Cell. (2014) 156:317–31. doi: 10.1016/j.cell.2013.12.010 24439385 PMC4076414

[108] Yao S Pang M Wang Y Wang X Lin Y Lv Y . Mesenchymal stem cell attenuates spinal cord injury by inhibiting mitochondrial quality control-associated neuronal ferroptosis. Redox Biol. (2023) 67:102871. doi: 10.1016/j.redox.2023.102871, 37699320 PMC10506061

[109] Doll S Proneth B Tyurina YY Panzilius E Kobayashi S Ingold I . ACSL4 dictates ferroptosis sensitivity by shaping cellular lipid composition. Nat Chem Biol. (2017) 13:91–8. doi: 10.1038/nchembio.2239, 27842070 PMC5610546

[110] Fleming JC Norenberg MD Ramsay DA Dekaban GA Marcillo AE Saenz AD . The cellular inflammatory response in human spinal cords after injury. Brain. (2006) 129:3249–69. doi: 10.1093/brain/awl296 17071951

[111] Brambilla R Bracchi-Ricard V Hu WH Frydel B Bramwell A Karmally S . Inhibition of astroglial nuclear factor kappaB reduces inflammation and improves functional recovery after spinal cord injury. J Exp Med. (2005) 202:145–56. doi: 10.1084/jem.20041918, 15998793 PMC2212896

[112] Siffrin V Radbruch H Glumm R Niesner R Paterka M Herz J . In vivo imaging of partially reversible th17 cell-induced neuronal dysfunction in the course of encephalomyelitis. Immunity. (2010) 33:424–36. doi: 10.1016/j.immuni.2010.08.018, 20870176

[113] Kwon BK Stammers AM Belanger LM Bernardo A Chan D Bishop CM . Cerebrospinal fluid inflammatory cytokines and biomarkers of injury severity in acute human spinal cord injury. J Neurotrauma. (2010) 27:669–82. doi: 10.1089/neu.2009.1080, 20038240

[114] Hellenbrand DJ Quinn CM Piper ZJ Elder RT Mishra RR Marti TL . The secondary injury cascade after spinal cord injury: an analysis of local cytokine/chemokine regulation. Neural Regen Res. (2024) 19:1308–17. doi: 10.4103/1673-5374.385849, 37905880 PMC11467934

[115] Zheng J Wu H Wang X Zhang G Lu J Xu W . Temporal dynamics of microglia-astrocyte interaction in neuroprotective glial scar formation after intracerebral hemorrhage. J Pharm Analysis. (2023) 13:862–79. doi: 10.1016/j.jpha.2023.02.007, 37719195 PMC10499589

[116] Chen J Zeng X Wang L Zhang W Li G Cheng X . Mutual regulation of microglia and astrocytes after Gas6 inhibits spinal cord injury. Neural Regen Res. (2025) 20:557–73. doi: 10.4103/NRR.NRR-D-23-01130, 38819067 PMC11317951

[117] Kwon HS Koh SH. Neuroinflammation in neurodegenerative disorders: the roles of microglia and astrocytes. Translational Neurodegeneration. (2020) 9:42. doi: 10.1186/s40035-020-00221-2, 33239064 PMC7689983

[118] Seegren PV Harper LR Downs TK Zhao XY Viswanathan SB Stremska ME . Reduced mitochondrial calcium uptake in macrophages is a major driver of inflammaging. Nature aging. (2023) 3:796–812. doi: 10.1038/s43587-023-00436-8, 37277641 PMC10353943

[119] Zhao C Rao JS Duan H Hao P Shang J Fan Y . Chronic spinal cord injury repair by NT3-chitosan only occurs after clearance of the lesion scar. Signal Transduct Target Ther. (2022) 7:184. doi: 10.1038/s41392-022-01010-1, 35710784 PMC9203793

[120] Sofroniew MV. Astrocyte barriers to neurotoxic inflammation. Nat Rev Neurosci. (2015) 16:249–63. doi: 10.1038/nrn3898, 25891508 PMC5253239

[121] Bracken MB Shepard MJ Collins WF Holford TR Young W Baskin DS . A randomized, controlled trial of methylprednisolone or naloxone in the treatment of acute spinal-cord injury. Results of the second national acute spinal cord injury study. N Engl J Med. (1990) 322:1405–11. doi: 10.1056/NEJM199005173222001, 2278545

[122] Assinck P Duncan GJ Hilton BJ Plemel JR Tetzlaff W. Cell transplantation therapy for spinal cord injury. Nat Neurosci. (2017) 20:637–47. doi: 10.1038/nn.4541, 28440805

[123] Fitch MT Silver J. CNS injury, glial scars, and inflammation: inhibitory extracellular matrices and regeneration failure. Exp Neurol. (2008) 209:294–301. doi: 10.1016/j.expneurol.2007.05.014, 17617407 PMC2268907

[124] Leal-Filho MB. Spinal cord injury: from inflammation to glial scar. Surg Neurol Int. (2011) 2:112. doi: 10.4103/2152-7806.83732, 21886885 PMC3162797

[125] Gage FH Temple S. Neural stem cells: generating and regenerating the brain. Neuron. (2013) 80:588–601. doi: 10.1016/j.neuron.2013.10.037, 24183012

[126] Young RA. Control of the embryonic stem cell state. Cell. (2011) 144:940–54. doi: 10.1016/j.cell.2011.01.032, 21414485 PMC3099475

[127] Nakamura M Okano H Toyama Y Dai HN Finn TP Bregman BS. Transplantation of embryonic spinal cord-derived neurospheres support growth of supraspinal projections and functional recovery after spinal cord injury in the neonatal rat. J Neurosci Res. (2005) 81:457–68. doi: 10.1002/jnr.20580, 15968644

[128] Kamada T Koda M Dezawa M Yoshinaga K Hashimoto M Koshizuka S . Transplantation of bone marrow stromal cell-derived Schwann cells promotes axonal regeneration and functional recovery after complete transection of adult rat spinal cord. J Neuropathol Exp Neurol. (2005) 64:37–45. doi: 10.1093/jnen/64.1.37, 15715083

[129] Parr AM Kulbatski I Zahir T Wang X Yue C Keating A . Transplanted adult spinal cord-derived neural stem/progenitor cells promote early functional recovery after rat spinal cord injury. Neuroscience. (2008) 155:760–70. doi: 10.1016/j.neuroscience.2008.05.042, 18588947

[130] Rosenzweig ES Brock JH Lu P Kumamaru H Salegio EA Kadoya K . Restorative effects of human neural stem cell grafts on the primate spinal cord. Nat Med. (2018) 24:484–90. doi: 10.1038/nm.4502, 29480894 PMC5922761

[131] Pang QM Chen SY Xu QJ Fu SP Yang YC Zou WH . Neuroinflammation and scarring after spinal cord injury: therapeutic roles of MSCs on inflammation and glial scar. Front Immunol. (2021) 12:751021. doi: 10.3389/fimmu.2021.751021, 34925326 PMC8674561

[132] Su Z Yuan Y Cao L Zhu Y Gao L Qiu Y . Triptolide promotes spinal cord repair by inhibiting astrogliosis and inflammation. Glia. (2010) 58:901–15. doi: 10.1002/glia.20972, 20155820

[133] Kabat M Bobkov I Kumar S Grumet M. Trends in mesenchymal stem cell clinical trials 2004-2018: is efficacy optimal in a narrow dose range? Stem Cells Transl Med. (2020) 9:17–27. doi: 10.1002/sctm.19-0202, 31804767 PMC6954709

[134] Huang Y Zheng Y Wang Q Qi C. Rolipram suppresses migration and invasion of human choriocarcinoma cells by inhibiting phosphodiesterase 4-mediated epithelial-mesenchymal transition. J Biochem Mol Toxicol. (2023) 37:e23363. doi: 10.1002/jbt.23363, 37020384

[135] Xia ML Xie XH Ding JH Du RH Hu G. Astragaloside IV inhibits astrocyte senescence: implication in Parkinson's disease. J Neuroinflammation. (2020) 17:105. doi: 10.1186/s12974-020-01791-8, 32252767 PMC7137443

[136] Hu X White K Olroyd AG DeJesus R Dominguez AA Dowdle WE . Hypoimmune induced pluripotent stem cells survive long term in fully immunocompetent, allogeneic rhesus macaques. Nat Biotechnol. (2024) 42:413–23. doi: 10.1038/s41587-023-01784-x, 37156915 PMC10940156

